# Pneumococcal community-acquired pneumonia in hospitalized adults in México 2018–2021: serotype distribution and clinical characteristics

**DOI:** 10.3389/fpubh.2026.1803418

**Published:** 2026-07-09

**Authors:** Juan Carlos Tinoco, Marivel Guereca, Jennifer Onwumeh-Okwundu, Yolanda Martinez-Lopez, Patricia Rodríguez-Zulueta, Verónica Aquino-Cruz, Mussaret B Zaidi, Samuel Navarro-Alvarez, Derek Daigle, Mohammad Ali, Marcelo Diaz, Carlos Andres Molina, Dania Abreu, Pia Marino, Mark A. Fletcher

**Affiliations:** 1 Hospital General, Servicios de Salud del Estado de Durango, Durango, Mexico; 2 RWE Epidemiology & Operations, Chief Medical Office, Pfizer, Collegeville, PA, United States; 3 Instituto de Investigación Cientifica, Universidad Juarez del Estado de Durango, Durango, Mexico; 4 Hospital General Dr. Manuel Gea Gonzalez, Mexico City, Mexico; 5 Hospital General O'Horan, Merida, Yucatan, Mexico; 6 Hospital General de Tijuana, Tijuana, Mexico; 7 US Medical Affairs, Vaccines and Antivirals, Pfizer, New York City, NY, United States; 8 Pfizer, Collegeville, New York City, New York; 9 Pfizer, Mexico City, Mexico; 10 Medical Scientific Liaison, Pfizer, Mexico City, Mexico; 11 Medical Affairs, Vaccines R&D, International Emerging Markets, Pfizer, Paris, France

**Keywords:** adult, community-acquired pneumonia, Mexico, *S. pneumoniae* serotypes, urine antigen detection

## Abstract

**Background:**

Community-acquired pneumonia (CAP) in adults represents a significant burden on the healthcare system in México, particularly among high-risk groups.

**Materials and methods:**

Adults hospitalized with chest X-ray-confirmed CAP (2018–2021) were prospectively enrolled across four cities in México (Mexico City, Durango, Mérida, and Tijuana). Urine samples were screened for *Streptococcus pneumoniae* (*S. pneumoniae*) using the BinaxNOW^®^ test and serotype-specific urine antigen detection (ssUAD) assays targeting 24 serotypes, and blood was obtained for pneumococcal culture.

**Results:**

Of the 1,275 patients screened for CAP, 389 had clinically or radiologically confirmed CAP (65.3% male; median age, 60 years; 39.3% aged ≥65 years). Pneumococcal CAP (pCAP) was identified in 12.1% of patients (47/389; 95% CI: 9.01–15.74). Based on CRB-65 scores, pCAP was more frequent among high-risk CAP patients (25.5%, 12/47) than among low- or moderate-risk CAP patients (10.2%, 35/342). Nevertheless, the 30-day mortality rate was 34.0% among pCAP patients versus 29.5% among *S. pneumoniae*-negative CAP patients. The most prevalent serotypes were 3 (13.0%), 6A (8.7%), and 19A (8.7%), and PCV20 would have covered 54.3% (25/46) of pCAP serotypes identified in this study.

**Conclusion:**

During the study period, CAP was associated with a substantial mortality burden among hospitalized adult patients in México, with pCAP more frequent among high-risk patients. These findings underscore the needs for enhanced diagnostic approaches and risk group-targeted adult pneumococcal vaccination programs.

## 1 Introduction

Adult community-acquired pneumonia (CAP) is associated with substantial morbidity and mortality worldwide (1), and *Streptococcus pneumoniae* (*S. pneumoniae*) is one of its leading causes (2). Determining the prevalence of disease-causing serotypes, their contribution to severe disease and their propensity to affect vulnerable populations, is critical for informed disease management and prevention (3–6). In Latin America, *S. pneumoniae* accounts for 35–40% of all hospitalized adult CAP cases (7–10). Prospective surveillance in Brazil among adults aged ≥50 years reported a CAP incidence of 20.1 cases per 10,000 person-years, with incidence rates increasing with age. Notably, 59.5% of serotyped pneumococcal CAP (pCAP) isolates corresponded to PCV20 serotypes (11). In México, pneumonia remains one of the leading causes of hospitalization and death among older adults, with a substantial proportion of the disease burden concentrated among individuals aged ≥65 years. In 2021, more than 33,000 cases of pneumonia were reported among individuals aged ≥65 years, a group at higher risk of severe disease and associated mortality (12, 13).

The clinical relevance of higher-valency vaccines is increasingly supported by global data. Urinary antigen detection assays have enhanced the identification of pneumococcal CAP and revealed that a substantial proportion of cases among adults are caused by serotypes covered by PCV13 and PCV20 in multiple countries with established pediatric PCV national immunization programs. Notably, PCV20 serotypes account for a considerably greater proportion of radiologically confirmed CAP cases compared to PCV13 serotypes alone (1). Furthermore, the persistence of certain PCV13 serotypes, together with the uncovering of non-PCV13 serotypes, has led to the introduction of higher-valency vaccines such as PCV15 and PCV20, which are designed to target remaining serotypes responsible for pneumococcal disease (14).

The National Immunization Program (NIP) of México has progressively introduced pneumococcal conjugate vaccines into the childhood immunization schedule, beginning with PCV7 in 2006, followed by PCV10 in 2010 and PCV13 in 2011 (15, 16). Under the NIP, children <5 years of age receive a three-dose series consisting of primary doses at 2 and 4 months of age, followed by a booster dose at 1 year of age (17). A single-dose PPSV23 program was introduced in 1993, targeting older and high-risk adults, and PCV13 vaccination was initiated in 2022 (18). For adults aged ≥60 years, the current recommendation is one dose of PCV13 followed by one dose of PPSV23 a year later. No catch-up programs are in place, and vaccination coverage in adults remains suboptimal (13). Table 1 summarizes the timeline of pneumococcal vaccination introduction in México. In México, the *Sistema Regional de Vacunas* laboratory surveillance program focuses on invasive pneumococcal disease in children, leaving a critical gap in serotype data among adults (10). México’s 2026 NIP recognizes older adults and individuals with risk factors as priority groups for the prevention of pneumococcal pneumonia, considering it a vaccine-preventable disease with a high public health impact. NIP recommendations for adults emphasize the need to update vaccination schedules based on current epidemiology and technologies, favoring conjugate vaccines for their higher immunogenicity and potential indirect benefits compared to polysaccharide-only options (19). PCV20 expands coverage to 20 serotypes in a single-dose option, including those relevant to the adult population in México. This simplifies vaccination and supports operational needs by eliminating the need for complex PCV + PPSV23 sequences, facilitating adult vaccination coverage (20). Although adult pCAP poses a considerable burden (12), *S. pneumoniae* surveillance of serotypes and antibiotic sensitivity is limited.

**Table 1:** Milestones in pneumococcal vaccination programs in México, 2000–2024 (46, 47).

Year	Milestone
2000	First pneumococcal conjugate vaccine (PCV7) authorized for use in children.
2004	Introduction of the 23-valent polysaccharide vaccine (PPSV23) for older adults.
2006	Introduction of the 7-valent pneumococcal conjugate vaccine (PCV7) in pediatric immunization programs in 58 states with lower rates of social development.
2007	Universal introduction of PCV7 in the National Immunization Program (NIP) for children under 1 year of age.
2010	Introduction of the 10-valent pneumococcal conjugate vaccine for children in the Mexican Institute for Social Security (IMSS).
2011	Introduction of the 13-valent pneumococcal conjugate vaccine in the NIP, replacing PCV7.
2024	Approval of PCV20 for pediatric (2 + 1 and 3 + 1 schedules) and adult use by the Committee of New Molecules, COFEPRIS.

The primary objective of this study was to identify the serotypes detected among adult pCAP patients confirmed by chest radiography using serotype-specific urine antigen detection (ssUAD) (see Section 2. Materials and Methods). Secondary objectives included identifying the major risk factors for CAP and estimating the proportion of CAP hospitalizations and deaths attributable to *S. pneumoniae* infection among all hospitalized adults. An exploratory objective was to estimate the proportion of pCAP among CAP patients.

## 2 Materials and methods

### 2.1 Study design and settings

This was a prospective cohort study conducted from November 2018 to July 2021 that included adults aged 18 years or older who were hospitalized with CAP in four cities in México, representing the North (Tijuana and Durango), Central (México City (Ciudad de México)), and South (Mérida) regions. Approximately 70% of the Mexican population is covered exclusively by public healthcare (21), and all participating centers were publicly funded, secondary-level hospitals serving underprivileged populations without private insurance.

### 2.2 Participants

All patients hospitalized at one of the four enrolling hospitals were evaluated for eligibility based on their medical history and clinical presentation, with CAP confirmed by chest radiography. Eligible patients were included after they provided informed written consent using the form approved by the ethics committee(s). As per the study protocol, physicians recorded clinical and radiological information on a standardized questionnaire and collected urine samples for *S. pneumoniae* detection using the BinaxNOW^®^ (Binax) test, followed by ssUAD. Information collected in the questionnaire included age, sex, smoking status, alcohol use, comorbidities, and influenza and pneumococcal vaccination history. Pneumonia severity was assessed using the CRB-65 index. Patient information was entered into a standardized database using SPSS. Participants were followed until discharge to record clinical outcomes.

### 2.3 Inclusion criteria

Patients were eligible for the study at any ward of one of the enrolling centers if they were aged 18 years or older and hospitalized with a history, clinical presentation, and chest radiography findings consistent with CAP. As intensive care unit admission was not included in the inclusion criteria, these details were not recorded and unavailable for analysis in this study. A CAP episode was characterized by the presence of two or more of the following clinical criteria: Fever (axillary temperature ≥38.0 °C) or hypothermia (axillary temperature <35.5 °C), measured by a healthcare provider; chills or rigors; pleuritic chest pain; cough with purulent sputum production; dyspnea or tachypnea; abnormal auscultatory findings suggestive of pneumonia; abnormal laboratory results, including leukocytosis (white blood cell count >15×10^9^ white blood cells/L or >15% bands) and elevated C-reactive protein (>3 times the upper limit of normal); and hypoxemia, defined as partial oxygen saturation (PO_2_) ≤ 60 mm Hg while breathing room air. Radiologically confirmed CAP was defined as chest X-ray findings consistent with pneumonia (i.e., pleural effusion, increased pulmonary density due to infection-like presence of alveolar infiltrates, bronchograms, etc.).

### 2.4 Exclusion criteria

Patients were excluded if they had a recent prior hospitalization (within 30 days for any admission diagnosis), were transferred from another hospital, or had been hospitalized for more than 48 hours. Previous enrollment in the study within 30 days before this presentation also resulted in exclusion. Patients were excluded if they had other severe conditions (medical or psychiatric) or a laboratory abnormality that, in the judgment of the investigator, would increase the risk associated with study participation or interfere with the interpretation of study results, thereby rendering the patient ineligible. Medical radiologists evaluated chest X-rays. If this evaluation was discordant with the physician’s opinion, the patient was excluded.

### 2.5 Laboratory procedures

At the investigational sites included in this adult CAP study, microbiologic analyses such as sputum culture or urinary BinaxNOW^®^ (Binax) testing were not routinely performed in patients. Routine peripheral venous blood cultures (approximately 10 mL) were obtained from all participants according to the standard procedures. Prior antibiotic exposure and the timing of specimen collection relative to treatment were not recorded. Although the four local hospitals in this study lacked serotyping facilities, pneumococcal isolates from blood cultures in Ciudad de México were sent to the National Reference Center (National Institute of Public Health, Cuernavaca, Morelos) for serotyping. Before starting standard-of-care antibiotic treatment, as determined by the clinical judgment of the attending physician, urine samples were collected from all participants (at least 30 mL) to perform the detection of urinary antigen using the Binax assay and two ssUAD assays, UAD1 and UAD2. UAD1 includes the detection of the PCV13 formulation serotypes. UAD2 identifies 11 serotypes, including the seven PCV20 non-PCV13 serotypes (8, 10A, 11A, 12F, 15B, 22F, and 33F) and four non-PCV20 serotypes (2, 9N, 17F, and 20). Blood culture sensitivity (0.74 to 0.75 but 0.24 among patients with antibiotic exposure) and specificity (0.78) in CAP have been estimated (22, 23) as well as for Binax (0.740 sensitivity and 0.972 specificity) (24) and ssUAD (0.922 to 0.97 sensitivity and 0.959 to 1.00 specificity) (25, 26). Urine samples were stored at −70 °C in a non-frost freezer until transport through World Courier to the Pfizer laboratory (New York, United States) for Binax and both ssUAD assays. It is important to note that ssUAD serotyping was attempted in all pCAP cases, and any cases without a final serotype assignment were due to technical limitations rather than selective sampling.

### 2.6 Control urine subjects

To determine the cutoff values for the diagnostic test performance of the ssUAD assays, 400 hospital-based control urine samples were collected (≥30 mL) from healthy Mexican adults. Voluntary participation in the ssUAD study was offered to patients attending any of the study centers who met the following eligibility criteria: (1) adults (≥18 years) and (2) provision of a signed and dated informed consent form. Investigational site staff members and their relatives were not eligible, nor were individuals directly involved in the conduct of the trial. Patients with suspected CAP or other respiratory infectious diseases, those with evidence of a documented concomitant infectious disease, or those residing in any long-term care facility were also excluded. Patients with fever, primary lung cancer, metastatic malignancy involving the lungs, or significant immunosuppressive conditions were also ineligible. Patients who had received any pneumococcal vaccination within the past 30 days were excluded. Patients with known bronchial obstruction or a history of post-obstructive pneumonia were not included; however, patients with chronic obstructive pulmonary disease were eligible, provided there was no exacerbation within the 3 months before enrollment. Thresholds for defining UAD assay positivity were set using nonparametric tolerance intervals to achieve at least 97% specificity per serotype based on the 400 urine samples collected from healthy controls (25, 26).

### 2.7 Statistical methods

Proportions were calculated by dividing the number of events by the population size. The proportion of *S. pneumoniae* serotypes, hospitalization rates, and mortality rates were expressed using percentages. All other binary and categorical variables—including the 30-day mortality rate, the presence of risk factors and comorbidities, different CAP etiologies, and previous vaccination status—were summarized using frequencies and percentages. *p*-values for the differences were computed using either the chi-squared test or Fisher’s exact test, as appropriate.

An event or episode of CAP was defined based on the WHO criteria as an acute infection of the lung parenchyma caused by *S. pneumoniae* contracted outside the healthcare setting, characterized clinically by the presence of two or more of the inclusion criteria described in the Methods section. The analysis groups included: (a) participants diagnosed with CAP (either clinically diagnosed or both clinically and radiologically confirmed) in whom *S. pneumoniae* testing was performed to detect ssUAD1- and ssUAD2-positive serotypes and (b) participants diagnosed with CAP (either clinically diagnosed or both clinically and radiologically confirmed) who were *S. pneumoniae-*positive but for whom the serotype could not be identified.

All statistical analyses were performed using Stata version 17.0.

### 2.8 Ethical considerations

The clinical study was designed, implemented, and reported according to the Harmonized Tripartite Guideline for Good Clinical Practice. The study was approved by the Committee on Ethics of Investigation of each participating institution.

## 3 Results

**Figure 1:**
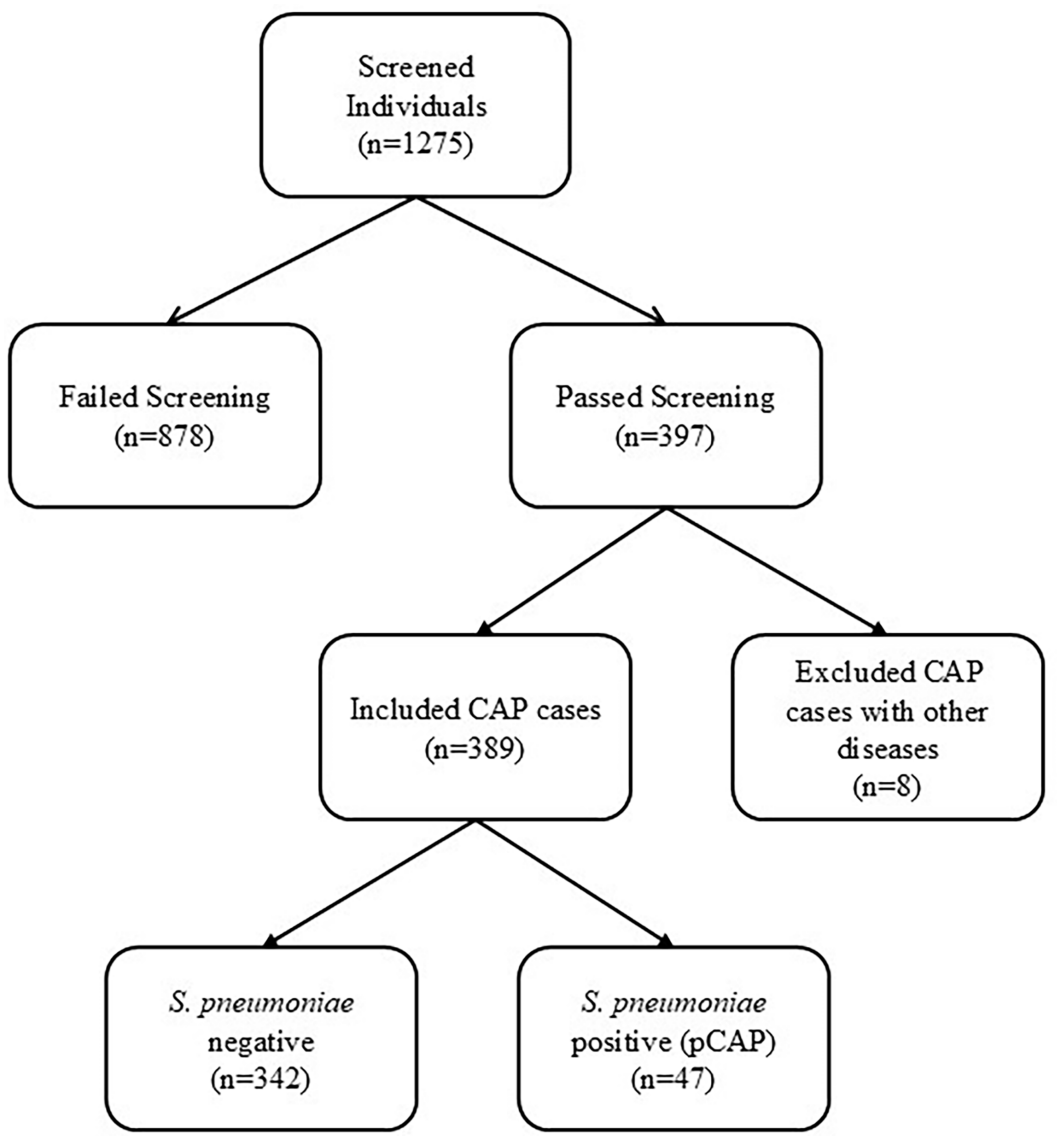
Study flow chart.

Of 1,275 screened individuals, CAP was confirmed in 389 patients based on clinical or combined clinical and radiological criteria. Among them, 47 pCAP patients (12.1%; 95% CI:9.01–15.74) tested positive for *S. pneumoniae* by Binax, ssUAD, or blood culture (Figure 1).

The median age of hospitalized CAP patients was 60 years, with 39.3% aged ≥65 years. Among the 47 pCAP patients, the median age was 56.5 years, with 40.4% aged ≥65 years. Pneumococcal vaccination prior to study enrollment was documented in only 1.5% of CAP patients (6/389), while influenza vaccination was reported in 15.2% (59/389).

A total of 389 hospitalized adult CAP patients were included in the analysis, of whom 47 (12.1%) were classified as pCAP and 342 (87.9%) as non-pCAP (Table 2). The proportion of pCAP cases varied by city, ranging from 8.5 to 31.9%, with the highest frequency observed in Durango. The two groups (pCAP vs. non-pCAP) did not differ significantly with respect to region (*p* = 0.18), sex (*p* = 0.58), age group (*p* = 0.38), or educational level (*p* = 0.15). Male patients comprised 61.7% of pCAP and 65.8% of non-pCAP cases. Regarding personal history, smoking was significantly more prevalent among pCAP patients (59.6%) compared to non-pCAP patients (31.6%; *p* = 0.0002). Similarly, a history of drug abuse was more common in the pCAP group (21.3% vs. 5.3%; *p* = 0.0006). In contrast, the prevalence of alcoholism did not differ significantly between the groups (36.2% vs. 25.2%; *p* = 0.11). Similarly, receipt of pneumococcal or influenza vaccines did not differ significantly between the groups (*p* = 0.54 and *p* = 0.42, respectively), although overall vaccination coverage was low in both groups.

**Table 2:** Demographics and clinical characteristics of pCAP and non-pCAP patients.

Characteristics		pCAP (*N* = 47)	Non-pCAP (*N* = 342)	*p*-value
		*n* (%)	*n* (%)	
Region	Ciudad de México	14 (29.8)	66 (19.3)	0.18
	Durango	15 (31.9)	160 (46.8)	
	Mérida	4 (8.5)	22 (6.4)	
	Tijuana	14 (29.8)	94 (27.5)	
Sex	Male	29 (61.7)	225 (65.8)	0.58
	Female	18 (38.3)	117 (34.2)	
Age group	18–49 years	16 (34.0)	90 (26.3)	0.38
	50–64 years	12 (25.5)	118 (34.5)	
	≥65 years	19 (40.4)	134 (39.2)	
Level of education	Illiterate	7 (14.9)	22 (6.4)	0.15
	Elementary school	19 (40.4)	153 (44.7)	
	Middle school	12 (25.5)	77 (22.5)	
	High school	7 (14.9)	47 (13.7)	
	Bachelor’s degree	2 (4.3)	43 (12.6)	
Personal history	Smoking history	28 (59.6)	108 (31.6)	0.0002
	Alcohol use	17 (36.2)	86 (25.2)	0.11
	Drug abuse	10 (21.3)	18 (5.3)	0.0006
Vaccination history	Received pneumococcal vaccine	1 (2.1)	5 (1.5)	0.54
	Received influenza vaccine	9 (19.2)	50 (14.6)	0.42
Initial condition	Fever	29 (61.7)	159 (46.5)	0.05
	Cough	33 (70.2)	152 (44.4)	0.0009
	Hypoxemia	37 (78.7)	228 (66.7)	0.10
Underlying diseases	Hypertension	12 (25.5)	159 (46.5)	0.04
	Diabetes mellitus	16 (34.0)	133 (38.9)	0.98
	Congestive heart failure	3 (6.4)	24 (7.0)	1.0000
	COPD	8 (17.0)	15 (4.4)	0.0014
Risk stratification CRB-65 score	Low risk	17 (36.2)	173 (50.6)	0.0076
	Moderate risk	18 (38.3)	134 (39.2)	
	High risk	12 (25.5)	35 (10.2)	
Evaluation	Cough	33 (70.2)	149 (43.6)	0.0006
	Altered state of consciousness	15 (31.9)	63 (18.4)	0.03
	Gastrointestinal abnormalities	11 (23.4)	40 (11.7)	0.03
	Confusion	17 (36.2)	66 (19.3)	0.01
	Breathing frequency >30	17 (36.2)	77 (22.5)	0.04
Discharge	Altered state of consciousness	6 (12.8)	19 (5.6)	0.10
	Pleural effusion	11 (23.4)	36 (10.5)	0.01
Clinical outcome	Improvement in clinical signs	29 (61.7)	238 (69.6)	0.19
	Voluntary discharge	2 (4.3)	9 (2.6)	
	Maximum therapeutic benefit	1 (2.1)	1 (0.3)	
	Death	15 (31.9)	93 (27.2)	
Final evaluation (30-day follow-up)*	Patients for final evaluation	31 (66.0)	250 (73.1)	-
	Cough with purulent sputum production	10 (32.3)	44 (17.6)	0.38
	Dyspnea or tachypnea	9 (29.0)	91 (36.4)	0.65
	Death after discharge	1 (3.2)	8 (2.3)	-

*P*-values were determined using the chi-squared test or Fisher’s exact test, as appropriate.*Final evaluation outcomes were assessed among patients available for 30-day follow-up: 31 patients with pCAP and 250 patients without pCAP. Percentages for final evaluation variables are calculated using these follow-up populations as denominators.

At hospital admission, pCAP patients more frequently presented with cough (70.2% vs. 44.4%; *p* = 0.0009) and hypoxemia (78.7% vs. 66.7%; *p* = 0.10), although hypoxemia did not reach statistical significance. Fever was observed more frequently in pCAP patients (61.7% vs. 46.5%; *p* = 0.05). Hypertension was less prevalent in pCAP patients (25.5% vs. 46.5%; *p* = 0.04). COPD was significantly more common in the pCAP group (17.0% vs. 4.4%; *p* = 0.0014). The prevalence of diabetes mellitus and congestive heart failure did not differ significantly.

With respect to risk stratification, pCAP patients were less likely to be classified as low risk according to the CRB-65 score (36.2% vs. 50.6%; *p* = 0.0076) and were more likely to have high-risk scores (25.5% vs. 10.2%), indicating greater severity at presentation. During hospitalization, pCAP patients more frequently exhibited cough (70.2% vs. 43.6%; *p* = 0.0006), confusion (36.2% vs. 19.3%; *p* = 0.0081), altered state of consciousness (31.9% vs. 18.4%; *p* = 0.03), breathing frequency >30 (36.2% vs. 22.5%; *p* = 0.04), and gastrointestinal abnormalities (23% vs. 11.7%; *p* = 0.03). Pleural effusion at discharge was also more common among pCAP patients (23.4% vs. 11%; *p* = 0.01). Death occurred in 31.9% of pCAP patients versus 27.2% of non-pCAP patients; however, clinical outcomes overall did not differ significantly between the groups (*p* = 0.19). Notably, 68.5% of CAP patients had underlying disease, with hypertension (44.0%) and diabetes mellitus (38.3%) being the most frequent. After 30-day follow-up, pCAP patients had higher rates of cough with purulent sputum production (32.3% vs. 17.6%; *p* = 0.38 and dyspnea or tachypnea was higher in non-pCAP patients (29.0%% vs. 36.4%; *p* = 0.65), although neither was significant. (Table 2).

Most ssUAD-positive patients were from Durango (36.7%, 11/30), and fewer patients were from Mérida (6.6%, 2/30). Ciudad de México and Tijuana had similar numbers of ssUAD-positive patients (30.0%, 9/30 and 26.7%, 8/30, respectively) (Supplementary Table S1).

Regarding clinical presentation, pCAP patients more frequently presented with fever (61.7% vs. 46.5%), cough (70.2% vs. 44.4%), and hypoxemia (78.7% vs. 66.7%) compared to *S. pneumoniae-*negative CAP patients. Based on CRB-65 scores, 25.5% (12/47) of pCAP patients were classified as high risk, compared to 10.2% (35/342) of non-pCAP patients classified as low or moderate risk. After the 30-day follow-up period, 117 of 389 CAP patients (30.1%) had died, including 16 of 47 pCAP patients (34.0%) (Table 2).

Among the 47 pCAP patients, serotype identification was obtained in all cases except one, which was Binax-positive only. A total of 40 samples tested positive by Binax, of which 24 were also positive by ssUAD. Of the eight Binax-negative samples, all were ssUAD-positive (Table 4). The most prevalent serotypes were 3 (13.0% 6/46), 6A (8.7%, 4/46), and 19A (8.7%, 4/46), all of which are PCV13 serotypes. PCV20 non-PCV13 serotypes identified two isolates of serotypes 10A and 22F (4.3%, 2/46 each) and single isolates of serotypes 8, 11A, and 12F (2.1%, 1/46 each). The only PPSV23 non-PCV20 serotypes identified were 9N (4.3%, 2/46) and 17F (22%, 1/46). By PCV formulation, 18 serotypes were PCV13, seven were PCV20 non-PCV13, and three were PPSV23 non-PCV20 (Table 3). Overall, 15 of 47 pCAP patients had no serotype identified using ssUAD targeting 24 serotypes (Table 4). Uniquely in Ciudad de México, blood culture isolates could be serotyped by Quellung reaction (Table 5). In total, four isolates (serotypes 6A / 6C, 9 N, 19A, and 19A) were obtained from patients with both Binax-and ssUAD-positive urine samples. Three isolates (serotypes 9 N, 23A, and 23B) were from patients who were Binax-positive but ssUAD-negative, and one isolate (serotype 15A) was from a patient who tested negative by both Binax and ssUAD.

**Table 3:** Serotype distribution among pCAP patients by urine BinaxNOW® or ssUAD, city, sex, and age group (*n* = 46).

Serotype	Total (*n* = 46)	Cities				Sex		Age group, years		
		Ciudad de México	Durango	Mérida	Tijuana	Male	Female	18–49	50–64	≥65
		(*n* = 14)	(*n* = 15)	(*n* = 4)	(*n* = 14)	(*n* = 29)	(*n* = 17)	(*n* = 16)	(*n* = 12)	(*n* = 18)
PCV13	**18 (39.1)**	**7 (50.0)**	**4 (26.7)**	**1 (25.0)**	**6 (42.8)**	**11 (37.8)**	**7 (38.9)**	**5 (31.4)**	**6 (50.0)**	**7 (36.8)**
3	6 (13.0)	1 (7.1)	2 (13.3)	–	3 (21.4)	4 (13.8)	2 (11.1)	3 (18.8)	1 (8.3)	2 (10.5)
6A	4 (8.7)	1 (7.1)	1 (6.7)	1 (25.0)	1 (7.1)	3 (10.3)	1 (5.6)	–	2 (16.7)	2 (10.5)
7F	3 (6.5)	1 (7.1)	1 (6.7)	–	1 (7.1)	1 (3.5)	2 (11.1)	1 (6.3)	1 (8.3)	1 (5.3)
19A	4 (8.7)	3 (21.4)	–	–	1 (7.1)	2 (6.9)	2 (11.1)	–	2 (16.7)	2 (10.5)
23F	1 (2.2)	1 (7.1)	–	–	–	1 (3.5)	–	1 (6.3)	–	–
PCV20 non-PCV13	**7 (15.2)**	**1 (7.1)**	**4 (26.7)**	**1 (25.0)**	**1 (7.1)**	**6 (20.9)**	**1 (5.6)**	**4 (25.1)**	**–**	**3 (15.8)**
8	1 (2.2)	–	–	1 (25.0)	–	1 (3.5)	–	1 (6.3)	–	–
10A	2 (4.3)	–	2 (13.3)	–	–	1 (3.5)	1 (5.6)	2 (12.5)	–	–
11A	1 (2.2)	–	1 (6.7)	–	–	1 (3.5)	–	–	–	1 (5.3)
12F	1 (2.2)	–	–	–	1 (7.1)	1 (3.5)	–	1 (6.3)	–	–
22F	2 (4.3)	1 (7.1)	1 (6.7)	–	–	2 (6.9)	–	–	–	2 (10.5)
Pairs	**2 (4.3)**	–	**1 (6.7)**	–	**1 (7.1)**	**2 (6.9)**	–	–	**2 (16.4)**	–
6A + 3	1 (2.2)	–	–	–	1 (7.1)	1 (3.5)	–	–	1 (8.3)	–
6A + 22F	1 (2.2)	–	1 (6.7)	–	–	1 (3.5)	–	–	1 (8.3)	–
PPSV23 non-PCV20	**3 (6.5)**	**1 (7.1)**	**2 (13.3)**	–	–	**2 (6.9)**	**1 (5.6)**	**1 (6.3)**	–	**2 (10.5)**
9N	2 (4.3)	1 (7.1)	1 (6.7)	–	–	2 (6.9)	–	1 (6.3)	–	1 (5.3)
17F	1 (2.2)	–	1 (6.7)	–	–	–	1 (5.6)	–	–	1 (5.3)

The bold values in this table are the total for each group serotype group by city, sex and age group.

**Table 4:** Serotype distribution among pCAP patients who tested positive by BinaxNOW® or ssUAD (*n* = 46).

BinaxNOW® status (*n* = 46)	ssUAD (+ve)		
	(*n* = 32)	Formulation (*n*)	Serotypes
BINAX (+ve) (*n* = 38)	ssUAD (+ve) (*n* = 24)	PCV13 (*n* = 16)	3 (*n* = 6), 6A (*n* = 5), 7F (*n* = 1), 19A (*n* = 4)
		PCV20 non-PCV13 (*n* = 5)	10A (*n* = 2), 11A (*n* = 1), 22F (*n* = 2)
		PPSV23 non-PCV20 (*n* = 3)	9N (*n* = 2), 17F (*n* = 1)
BINAX (−ve) (*n* = 8)	ssUAD (+ve) (*n* = 8)	PCV13 (*n* = 5)	3 (*n* = 1), 6A (*n* = 1), 7F (*n* = 2), 23F (*n* = 1)
		PCV20 non-PCV13 (*n* = 3)	8 (*n* = 1), 12F (*n* = 1), 22F (*n* = 1)

There were two ssUAD-positive pairs: One with serotypes 6A and 22F, and another with serotypes 3 and 6A.

**Table 5:** Serotype distribution among adult pCAP patients from Ciudad de México (*n* = 14).

Urine	Urine	Blood culture
BinaxNOW® result	ssUAD result	Quellung result
Binax positive	3	Culture negative
Binax positive	6A	6C
Binax positive	9 N	9 N
Binax positive	19A	19A
Binax positive	19A	19A
Binax positive	19A	Culture negative
Binax positive	ssUAD negative	9 N
Binax positive	ssUAD negative	23A
Binax positive	ssUAD negative	23B
Binax positive	ssUAD negative	Culture negative
Binax negative	7F	Culture negative
Binax negative	23F	Culture negative
Binax negative	22F	Culture negative
Binax negative	ssUAD negative	15A

Serotype distribution varied by age group and city. Among patients aged 18–49 years, serotype 3 was most frequently identified (18.8%). Among patients aged ≥65 years, serotypes 3, 6A, 19A, and 22F were identified with equal frequency. Among patients aged 50–64 years, serotypes 6A (16.7%) and 19A (16.7%) were most frequently isolated. Serotype 19A was predominant in Ciudad de México (21.4%), while serotype 3 was most frequently identified in Durango (13.3%) and Tijuana (21.4%). By sex, serotypes 3 (13.8%) and 6A (10.3%) were more common in male individuals than in female individuals. Serotypes 23F, 8, 11A, 12F, 22F, and 9N were identified exclusively in male individuals (Table 3).

## 4 Discussion

In the present study, pCAP was confirmed in 12.1% of hospitalized CAP patients across four geographically diverse Mexican cities. This proportion is lower than that reported in some Latin American studies, which ranged from 24% in Argentina (from 1997 to 1998, where the childhood PCV NIP began in 2012) (27, 28) to 78% in a multinational study (published in 2000, including the countries of México, Chile, Argentina, Uruguay, and Brazil, and conducted before any initial introduction of pediatric PCV7 programs) (28). By contrast, infants in México have received pneumococcal conjugate vaccines through the childhood NIP since 2006, which could have contributed to substantial indirect protection in unvaccinated adults in the community (29, 30). For instance, in the USA, between 2014 and 2016—approximately 15 years after the introduction of the PCV7 pediatric NIP (2000) and 6 years after the introduction of the PCV13 NIP (2010)—a similar methodology demonstrated that 13% of hospitalized adult CAP patients had pneumococcal pneumonia (31). As noted in the Materials and methods section, neither prior antibiotic exposure nor the timing of blood culture relative to treatment was recorded, which is a limitation of this study. In addition, the COVID-19 pandemic, which overlapped with the study period (2018–2021), may have reduced pneumococcal co-detection due to competitive viral suppression of bacterial pathogens, delayed hospital presentations, and disruptions to diagnostic workflows. These factors likely contributed to an underestimation of the true pCAP burden rather than reflecting a genuinely lower prevalence in this population (32).

The overall mortality rate in this study was high, with a total of 34.0% (16/47) among pCAP patients and 30.1% (117/389) among all CAP patients. By comparison, a 2013 retrospective analysis from México reported a mortality rate of 16% among hospitalized pneumonia patients (33). Although ICU admission details were unavailable for analysis in this study, the substantially higher mortality observed here is consistent with a more severely ill cohort, as reflected in the high proportion of CRB-65 high-risk patients and the strain on healthcare systems during the COVID-19 pandemic. During 2020–2021, approximately one-third of excess mortality in México was attributed to respiratory infections (34), and documented disruptions included reduced access to diagnostics and increased use of antibiotics associated with empirical therapy (35, 36). Indiscriminate antibiotic use, combined with dexamethasone administration during this period, may have contributed to culture-negative pneumonia cases and worse clinical outcomes. Indiscriminate antibiotic use, combined with delays in hospital admission and treatment due to telemedicine-related triage, hospital congestion, and insufficient adherence to CAP treatment guidelines because of staff and caregiver shortages, may have contributed to the high mortality observed in our setting (32). For instance, it was documented that 154 health policies across healthcare institutions were uncoordinated and heterogeneous, leading to inequalities in access to care (37). In addition, possible co-infections may have impacted survival rates (38). Formal attribution of mortality to pneumococcal disease specifically was outside the scope of this descriptive study; future studies should prospectively collect ICU admission data and apply standardized severity stratification to enable more granular mortality analysis.

The risk factor profile of pCAP patients revealed several clinically meaningful differences compared to non-pCAP patients. Smoking history was significantly more prevalent among pCAP patients (59.6% vs. 31.6%; *p* = 0.0002), consistent with well-established evidence that cigarette smoking impairs mucociliary clearance, disrupts respiratory epithelial integrity, and suppresses local innate immune defenses, collectively increasing susceptibility to pneumococcal colonization (39). Similarly, drug abuse was markedly more frequent in the pCAP group (21.3% vs. 5.3%; *p* = 0.0006), likely reflecting immunosuppression, poor nutritional status, and reduced healthcare-seeking behavior, which may facilitate progression from colonization to pneumonia (40). These behavioral risk factors are particularly relevant in the study population, which consisted of publicly insured, lower-socioeconomic status adults (41).

COPD was significantly more prevalent among pCAP patients (17.0% vs. 4.4%; *p* = 0.0014), consistent with the recognized role of COPD as a major risk factor for pneumococcal pneumonia. Chronic airflow obstruction and associated structural lung damage create a permissive environment for bacterial proliferation, and COPD patients have impaired opsonization and phagocytic responses that reduce clearance of encapsulated organisms such as *S. pneumoniae* (42). This finding further supports the targeted inclusion of COPD patients in adult pneumococcal vaccination programs, which is already recommended in several national guidelines but remains poorly implemented in México. Hypertension was less prevalent in pCAP patients (25.5% vs. 46.5%; *p* = 0.04), a finding that likely reflects a competing comorbidity effect.

The higher frequency of confusion (36.2% vs. 19.3%; *p* = 0.01), altered state of consciousness (31.9% vs. 18.4%; *p* = 0.03), and breathing rate >30 (36.2% vs. 22.5%; *p* = 0.04) among pCAP patients during hospitalization further corroborates the severity gradient associated with pneumococcal etiology. The higher rates of cough with purulent sputum and hypoxia at 30-day follow-up might suggest that pCAP is associated with a more prolonged and complicated recovery, with greater residual respiratory morbidity among survivors—findings with important implications for healthcare resource utilization and post-discharge management.

With respect to serotype distribution, serotype 3 was the most prevalent (13.0%), followed by 6A and 19A (8.7% each), all of which are PCV13 serotypes. These findings are broadly consistent with data from the EGNATIA study in Greece (43), where serotypes 3, 19A, and 23F predominated among hospitalized adults, and with a Spanish study using ssUAD that reported PCV13 serotypes as responsible for 14.1% of CAP cases in immunocompetent adults (44). The majority of serotypes identified in our study are covered by PCV20, including PCV13 serotypes and PCV20 non-PCV13 serotypes such as 22F, 10A, 8, 11A, and 12F identified in our cohort. This is particularly relevant given that México’s current adult NIP recommends PCV13, and our data suggest that a transition to PCV20 could extend serotype coverage in the adult population. The identification of two serotypes in individual patients, such as 6A + 22F and 3 + 6A, likely reflects true pneumococcal co-colonization or co-infection, a phenomenon that has been documented in ssUAD-based studies (45).

Serotype identification was achieved in 63.8% (30/47) of pCAP cases (Table 4), where in two pCAP episodes serotype pairs were identified (Table 3) leading to an overall identification rate of 68.1% (32/47). The 31.9% of cases without a serotypes most likely represents the limits of the current 24-serotype ssUAD, as well as inhibitory substances in urine samples that could affect assay performance, and specimens collected after antibiotic initiation, which are all recognized limitations of antigen-based serotyping. Given that ssUAD targets a subset of non-bacteremic CAP serotypes and that certain uncovered are associated with disease, some challenges with antigen-based serotyping can be expected.

Vaccination coverage in this study was strikingly low, with only 1.5% (6/389) of patients having received a pneumococcal vaccine prior to enrollment. This is consistent with the broader context of adult immunization in México, where no catch-up programs exist, awareness of adult vaccination recommendations is limited, and access through publicly funded centers remains inconsistent. The absence of antimicrobial susceptibility data in this study represents an additional limitation; future surveillance should integrate susceptibility testing alongside serotyping to inform both vaccination and treatment policies.

The healthcare infrastructure in México has pre-existing challenges that were highlighted during the pandemic, and lack of access to proper treatment may have contributed to the high mortality observed in this study. It was documented that health policies across healthcare institutions were uncoordinated and heterogeneous, leading to inequalities in access to care (37). Finally, all participating centers were publicly funded secondary-level hospitals; therefore, the findings may not be fully generalizable to patients seeking care in private healthcare facilities, who may differ in clinical and socioeconomic characteristics. Approximately 70% of the Mexican population relies on public healthcare (21), indicating that our findings are representative of the majority of the burden-bearing population.

## 5 Conclusion

This study provides serotype-level evidence on pneumococcal CAP among hospitalized adults across four regions in México, which is critical for evaluating the potential impact of higher-valency pneumococcal vaccines in this population. The predominance of PCV13 serotypes, along with the presence of additional PCV20 non-PCV13 serotypes, supports consideration of PCV20 for inclusion in México’s adult immunization program. The high 30-day mortality observed, combined with a risk factor profile dominated by smoking, COPD, and drug abuse, underscores the severity of pCAP in publicly insured populations and highlights the need for improved diagnostic capacity, adherence to CAP management guidelines, and expanded targeted vaccination programs.

Several limitations should be noted: The descriptive study design precluded multivariable analysis, microbiological serotype confirmation was achieved in 63.8% of pCAP cases, ICU admission and detailed severity score beyond CRB-65 were not available, and the COVID-19 pandemic likely influenced both disease patterns and diagnostic yield during part of the study period. Future prospective studies integrating antimicrobial susceptibility testing, standardized severity scoring, and post-pandemic surveillance will be critical to building on these findings and informing evidence-based pneumococcal vaccination policies in México and other low- and middle-income settings.

## Data Availability

The original contributions presented in the study are included in the article/Supplementary material, further inquiries can be directed to the corresponding author.
