# Supplementary material for: Pneumococcal community-acquired pneumonia in hospitalized adults in México 2018–2021: serotype distribution and clinical characteristics
Source: Front Public Health. 2026 Jul 9;14:1803418. doi: 10.3389/fpubh.2026.1803418 (PMC13391570; doi:10.3389/fpubh.2026.1803418)
Supplement: Supplementary file 1 [file Table_1.docx]

Supplementary Tables

Table S1. Characteristics of the pCAP patients by urine BinaxNow® or ssUAD results (N=46)

| **Characteristics** | | **BINAX (+ve)**  **and**  **ssUAD (+ve)**  **(N=22)** | **BINAX (-ve)**  **and**  **ssUAD (+ve)**  **(N=8)** | **BINAX (+ve) and**  **ssUAD (-ve)**  **(N=16)** |
| --- | --- | --- | --- | --- |
|  | | n (%) | n (%) | n (%) |
| Region | México City | 7 (31.8) | 2 (25.0) | 4 (25.0) |
|  | Durango | 8 (36.4) | 3 (37.5) | 4 (25.0) |
|  | Mérida | 1 (4.5) | 1 (12.5) | 2 (12.5) |
|  | Tijuana | 6 (27.3) | 2 (25.0) | 6 (37.5) |
| Gender | Male | 15 (68.2) | 6 (75.0) | 8 (50.0) |
|  | Female | 7 (31.8) | 2 (25.0) | 8 (50.0) |
| Age group | 18 – 49 years | 6 (27.3) | 4 (50.0) | 6 (37.5) |
|  | 50 – 64 years | 6 (27.3) | 2 (25.0) | 4 (25.0) |
|  | ≥65 years | 10 (45.5) | 2 (25.0) | 6 (37.7) |
| Education | Illiterate | 4 (18.2) | 2 (25.0) | 1 (6.2) |
|  | Elementary School | 9 (40.9) | 2 (25.0) | 8 (50.0) |
|  | Middle School | 5 (22.7) | 2 (25.0) | 4 (25.0) |
|  | High School | 3 (13.6) | 2 (25.0) | 2 (12.5) |
|  | Bachelor | 1 (4.5) | 0 (0.0) | 1 (6.2) |
| Personal history | Smoking | 14 (63.6) | 6 (75.0) | 8 (50.0) |
|  | Alcohol use | 8 (36.4) | 5 (62.5) | 3 (18.8) |
|  | Drug Abuse | 7 (31.8) | 1 (12.5) | 2 (12.5) |
| Vaccination history | Received pneumococcal vaccine | 0 (0.0) | 0 (0.0) | 0 (0.0) |
|  | Received flu vaccine | 5 (22.7) | 2 (25.0) | 1 (6.2) |
